# *In vivo* MRI Characterization of Progressive Cardiac Dysfunction in the mdx Mouse Model of Muscular Dystrophy

**DOI:** 10.1371/journal.pone.0028569

**Published:** 2012-01-03

**Authors:** Daniel J. Stuckey, Carolyn A. Carr, Patrizia Camelliti, Damian J. Tyler, Kay E. Davies, Kieran Clarke

**Affiliations:** 1 Cardiac Metabolism Research Group, Department of Physiology, Anatomy and Genetics, University of Oxford, Oxford, United Kingdom; 2 Heart Science Centre, National Heart and Lung Institute, Imperial College, London, United Kingdom; 3 Medical Research Council Functional Genomics Unit, Department of Physiology, Anatomy and Genetics, University of Oxford, Oxford, United Kingdom; Medical College of Georgia, United States of America

## Abstract

**Aims:**

The mdx mouse has proven to be useful in understanding the cardiomyopathy that frequently occurs in muscular dystrophy patients. Here we employed a comprehensive array of clinically relevant *in vivo* MRI techniques to identify early markers of cardiac dysfunction and follow disease progression in the hearts of mdx mice.

**Methods and Results:**

Serial measurements of cardiac morphology and function were made in the same group of mdx mice and controls (housed in a non-SPF facility) using MRI at 1, 3, 6, 9 and 12 months after birth. Left ventricular (LV) and right ventricular (RV) systolic and diastolic function, response to dobutamine stress and myocardial fibrosis were assessed. RV dysfunction preceded LV dysfunction, with RV end systolic volumes increased and RV ejection fractions reduced at 3 months of age. LV ejection fractions were reduced at 12 months, compared with controls. An abnormal response to dobutamine stress was identified in the RV of mdx mice as early as 1 month. Late-gadolinium-enhanced MRI identified increased levels of myocardial fibrosis in 6, 9 and 12-month-old mdx mice, the extent of fibrosis correlating with the degree of cardiac remodeling and hypertrophy.

**Conclusions:**

MRI could identify cardiac abnormalities in the RV of mdx mice as young as 1 month, and detected myocardial fibrosis at 6 months. We believe these to be the earliest MRI measurements of cardiac function reported for any mice, and the first use of late-gadolinium-enhancement in a mouse model of congenital cardiomyopathy. These techniques offer a sensitive and clinically relevant *in vivo* method for assessment of cardiomyopathy caused by muscular dystrophy and other diseases.

## Introduction

Duchenne muscular dystrophy (DMD) is a progressive muscle wasting disease that affects approximately one in 3000 males [1], [2]. The absence of functional dystrophin restricts interaction and signal transduction between the cytoskeleton and the extracellular matrix in both skeletal muscle cells and cardiomyocytes [2], [3]. Advances in the treatment of respiratory failure in DMD patients has increased their life expectancy, but has resulted in many patients developing cardiomyopathy [2]. In the dystrophic heart, cytoskeleton dysfunction can lead to cell membrane rupture, cardiomyocyte necrosis and replacement of contractile myocardium with fibrotic tissue. This increases wall stress, reduces cardiac function and can lead to heart failure [2], [4]. Current treatments, including ACE inhibitors and β-blockers, slow disease progression, but the disease remains incurable.

The mdx mouse model of muscular dystrophy has been extensively studied [5], [6], [7], [8], [9], [10], [11], [12], [13], [14], [15] and, although exhibiting a mild form of muscular dystrophy, has been useful for studying pathological mechanisms, disease progression and therapy [4], [5], [6], [7], [8], [9], [10], [11], [12], [13], [14], [16], [17], [18], [19]. Novel gene, antisense oligonucleotide and cell therapies have been used to increase levels of dystrophin and restore skeletal and cardiac muscle function in the mdx mouse [4], [6], [11], [16], [19]. Antisense oligonucleotide therapy, already tested in patients, has shown promise for increasing synthesis of functional dystrophin [17], [20]. The development of non-invasive imaging methods that can provide accurate characterization of the efficacy of new therapeutic strategies in mice and humans will help translate such important therapies to the clinic.

Cardiac magnetic resonance imaging and echocardiography have been used to identify increased end systolic volumes and reduced ejection fractions in the right and left ventricles of muscular dystrophy patients [21], [22], [23]. Late gadolinium-enhanced (LGE) MR imaging has been used to identify fibrotic or inflamed tissue in the intraventricular septum, RV insertion points and anterolateral wall of the left ventricle [21], [22], [24], [25], [26], [27], [28]. Importantly, prognosis and cardiac function are poorer in DMD patients who have late gadolinium enhancement [24], [28].

In the mdx mouse, standard measures of left ventricular function, including ejection fraction, end systolic volume and wall thickening, do not become abnormal until 9 to 11 months of age [8], [13]. However, MRI has identified early abnormalities in cardiac wall strain and torsion [7], as well as right ventricular dysfunction at 6–8 months [6], [13]. Imaging studies of cardiac function in the mdx mouse have focused on mice at one specific age, or imaged different groups of mice at different ages, the earliest time point being ∼5-months [5], [6], [7], [8], [12], [13]. Here we serially imaged the same group of male mdx mice and littermate male wild type controls using MRI at 1 month after birth and at four further timepoints up to 1 year. We assessed left and right ventricular systolic function, diastolic function and response to dobutamine stress. Further, we report the first use of late gadolinium enhancement to identify regions of myocardial fibrosis in mdx mice. *In vivo* measures of fibrosis correlated with cardiac function, and may offer a sensitive and clinically relevant *in vivo* method for assessing the efficacy of experimental treatments for muscular dystrophy.

## Methods

The study conformed to EU Directive 2010/63/EU and was approved by The University of Oxford Animal Ethics Review Committees and the Home Office (London, UK) under Project License 30/2278. Ten male mdx and 12 male wild type C57Blk10 mice were housed in a non-SPF (specific pathogen free) facility, had free access to food and water and underwent serial cardiac MRI at 1, 3, 6, 9 and 12 months of age.

### Cardiac cine-MRI

Cardiac cine-MRI was performed as described [29], [30]. Briefly, mice were anesthetized with 1.5% isoflurane in O_2_ and positioned supine in a purpose-built cradle. ECG electrodes were inserted into the forepaws and a respiration loop was taped across the chest. The cradle was lowered into a vertical-bore, 11.7 T MR system (Magnex Scientific, Oxon, United Kingdom) with a 40 mm birdcage coil (Rapid Biomedical, Würzburg, Germany) and a Bruker console running Paravision 2.1.1 (Bruker Medical, Ettlingen, Germany). A stack of contiguous 1 mm thick true short-axis ECG and respiration-gated cine-FLASH images (TE/TR 1.43/4.6 ms; 17.5° pulse; field of view 25.6×25.6 mm; matrix size 256×256; voxel size 100×100×1000 µm; 20 to 30 frames per cardiac cycle) were acquired to cover the entire left and right ventricles of 3 to 12-month-old mice. For 1-month-old mice, a 28 mm birdcage coil (Rapid Biomedical) was used, with slice thickness 0.75 mm, field of view 19.2×19.2 mm; matrix size 192×192; voxel size 100×100×750 µm. Long-axis two-chamber and four-chamber images were also acquired.

### High temporal resolution cine-MRI

High temporal resolution cine-MRI was performed in a single mid LV short axis slice as described [30]. Briefly, ECG gated cine-FLASH images (TE/TR 0.86/2.4 ms; 60° pulse; field of view 25.6×25.6 mm; matrix size 128×128; voxel size 200×200×1000 µm; 40 to 60 frames per cardiac cycle) were acquired over two consecutive cardiac cycles providing data throughout diastole to determine peak ejection and filling rates.

### Late gadolinium-enhanced MRI

Late gadolinium-enhanced MRI (LGE-MRI) was performed to cover the entire LV at 10 to 25 min after intra-peritoneal injection of 0.5 mmol/kg Gd-DTPA using the same cardiac and respiratory gated, gradient echo cine-FLASH sequence (TE/TR 1.43/4.6 ms; ∼60° pulse; field of view 25.6×25.6 mm; matrix size 256×256), but with increased flip angle to saturate signal in the normal myocardium. This method is similar to that validated by Protti *et al* [31].

### Dobutamine stress MRI

Dobutamine stress testing was performed in 1, 3 and 6-month-old mice. An intra-peritoneal injection of 1.5 mg/kg of dobutamine was given whilst mice were still positioned in the scanner. Heart rate typically increased within 2 minutes of the injection, stabilized at 5 minutes and remained elevated for over 30 minutes. Once the heart rate had stabilized, 3 representative cine-FLASH MR images (basal, mid LV and apical) were acquired in the same locations as prior to dobutamine. Changes in volumes and ejection fraction are reported as the sum of these 3 slices. The entire *in vivo* imaging protocol was performed in approximately 70 minutes. Stress testing was not performed in 9 and 12 month mice as it caused mortality in older mice (DJS-unpublished).

### MRI data analysis

Image analysis was performed using ImageJ (NIH Image, Bethesda, MD). Left ventricular mass, volumes and ejection fraction were calculated as described [32]. Right ventricular volumes were calculated from the same stack of short axis cine images, but 2–3 extra slices at the base of the heart were required to cover the entire RV, as described by Wiesmann et al [33]. Wall thickness was measured at end diastole and end systole in the interventricular septum and the LV free wall in a mid-LV slice. LV ejection and filling rates were calculated from chamber volume measurements made in every frame of the high temporal resolution cine images using a semi-automated function in ImageJ [30]. Rates were calculated as the difference in volume over 3 consecutive frames divided by the time to acquire the frames as described [30].

*In vivo* late gadolinium-enhanced MR images were thresholded to two standard deviations above the mean signal intensity from remote tissue. Location and extent of hyper-enhancement were manually assessed and segmented.

### Histological analysis

After 1 and 15 months, mice were sacrificed by cervical dislocation, hearts were excised, perfused with saline and frozen in TissueTek OCT. Cryosections (10 µm) were cut and stained for collagen using sirius red.

### Data and statistical analysis

Results are shown as mean ± standard error. Differences were considered significant at *p*<0.05 using 2 way ANOVA.

## Results

### Serial measurements of resting cardiac morphology and function

**Figure 1:**
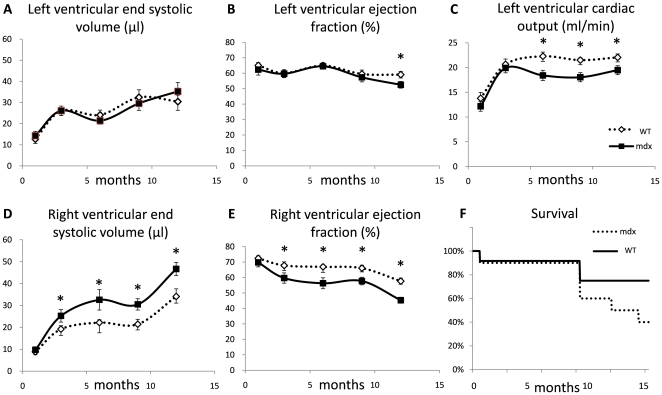
Serial measurements of cardiac function and mortality. *A–E;* Graphs of left and right ventricular morphology and function measured *in vivo* in mdx and WT mice from 1 to 12 months. *F;* Kaplein-Meier graph of mouse survival during the study indicating earlier mortality in mdx mice.

Mice were generally healthy and active throughout the study. However, after 9 months, the mortality in mdx mice increased, with 2 mice dying under anaesthesia and 3 more dying in the next 2 months (Figure 1). Mdx mice had increased body mass at 3, 6 and 9 months compared with wild type (WT) controls (Table 1). Heart rates under anaesthesia were similar in mdx and control mice. However, anaesthetic levels during imaging were adjusted to maintain heart rate at ∼500 bpm, potentially masking pathological differences.

**Table 1:** Left and right ventricular cardiac morphology and function.

		1 month	3 months	6 months	9 months	12 months
**Body mass (g)**	Con	14±1	29±2	31±2	32±1	35±1
	mdx	14±1	31±2	33±2	36±1*	36±1
**Heart mass∶body mass**	con	3.7±1	3.4±1	3.7±1	3.9±1	3.9±1
**ratio (×10^−3^ )**	mdx	3.9±1	3.5±1	3.5±1	3.2±1*	3.8±1
**Heart rate (bpm)**	con	438±11	527±17	503±10	464±4	519±16
	mdx	421±17	520±12	497±12	469±12	505±11
**Left ventricle**						
End diastolic volume (ul)	con	36±3	65±2	68±4	78±4	73±4
	mdx	37±3	65±3	60±4	68±4	74±6
End systolic volume (ul)	con	13±1	26±1	24±2	32±4	30±3
	mdx	14±2	26±2	21±2	30±3	35±4
Stroke volume (ul)	con	23±2	39±2	44±2	46±1	42±2
	mdx	23±2	38±2	39±3	38±2*	38±3
Ejection fraction (%)	con	65±2	60±2	65±2	60±3	59±2
	mdx	62±3	60±2	64±1	57±3	53±2*
Cardiac output	con	14±1	21±1	22±1	22±1	22±1
	mdx	12±1	20±1	18±1*	18±1*	19±1*
Peak filling rate	con	-	444±40	479±68	566±66	376±70
	mdx	-	375±40*	312±37*	574±121	379±70
Peak ejection rate	con	-	−314±9	−343±35	−411±42	−343±22
	mdx	-	−267±14*	−329±31*	−397±46	−290±17
Mass (mg)	con	52±4	99±3	116±2	125±4	135±4
	mdx	54±5	109±4	116±5	118±4	135±3
**Wall thickness**						
Septum at diastole (mm)	con	0.84±1	0.95±1	1.13±1	1.04±1	1.20±2
	mdx	0.84±1	0.88±1	1.17±2	1.03±1	1.26±1
Free wall at diastole (mm)	con	0.72±3	0.88±3	0.97±4	0.90±3	1.04±4
	mdx	0.70±3	0.86±3	1.07±4	0.96±3	1.05±5
**Right ventricle**						
End diastolic volume (ul)	con	32±2	60±2	67±2	62±3	79±6
	mdx	23±3	62±3	72±5	72±3	86±5
End systolic volume (ul)	con	9±1	19±2	22±2	21±2	34±3
	mdx	9±1	25±3*	33±5*	30±3*	47±3*
Stroke volume (ul)	con	23±1	40±2	45±1	41±1	45±3
	mdx	22±2	37±2	39±2	41±2	39±3
Ejection fraction (%)	con	72±2	68±2	67±1	66±2	57±2
	mdx	71±3	60±3*	56±3*	58±2*	45±2*

Results are presented as means ± SEM.

**Figure 2:**
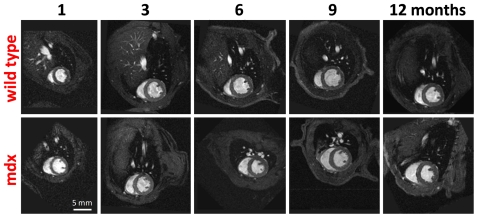
Cardiac MRI. *In vivo* short axis cine-MR images of the same wild type and mdx mouse imaged at 1, 3, 6, 9 and 12 months.

Cine-MRI was used to measure left and right ventricular morphology and function in mdx and WT mice from 1 to 12 months of age. Good quality images were obtained even from the smallest of the 1 month old mice weighing less than 10 g (Figure 2).

No alterations to left ventricular mass, septal and free wall thickness and thickening, end diastolic volume (EDV), end systolic volume (ESV), stroke volume (SV) or ejection fraction (EF) were identified in mdx mice before 9 months (Figure 1, Table 1). Cardiac output (CO) was lower in mdx mice at 6 and 9 months. SV was reduced at 9 months, but not at 12 months. However, there was a significant decrease in ejection fraction by 12 months in mdx mouse hearts. Measurements of rate of change in LV volume during contraction and relaxation using high temporal resolution cine-MRI, showed that peak ejection and peak filling rates were also lower in 3 and 6 month old mdx mice (Table 1).

Right ventricular ESV was higher and EF was lower in mdx mice by 3 months, a difference that was maintained at 6, 9 and 12 months (Figure 1, Table 1).

### Serial measurements of cardiac function under dobutamine stress

**Figure 3:**
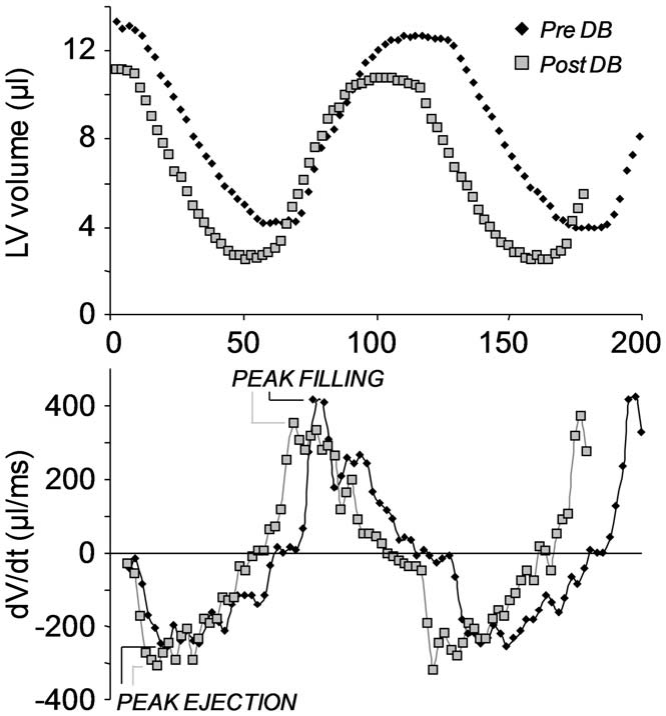
Left ventricular filling and ejection rates in control mouse. Time-volume curve *(upper)* and rate of change of volume curve *(lower)* acquired before and after bolus injection of 1.5 mg/kg dobutamine. The dobutamine induced an increase in peak ejection rate, but a reduction in peak filling rate, with lengthening of the high velocity filling phase. Mdx and control mice showed similar responses.

Administration of dobutamine to 1, 3 and 6-month-old WT mice increased heart rate by ∼20% in 1-month-old mice, although the increase became less with age (Table 2). Left ventricular EDV and ESV were reduced and EF was increased in response to dobutamine. A similar response was observed in mdx mice (Table 2). Septal and free wall thickness and thickening were increased to a similar degree in mdx and WT mice at each time point. In both mdx and WT mice, peak ejection rates increased under dobutamine stress, whereas peak filling rates were reduced (Figure 3, Table 2).

**Table 2:** Left and right ventricular cardiac function under dobutamine stress.

% change with dobutamine		1 month	3 months	6 months
**Left ventricle**				
End diastolic volume	con	−12±4	−21±3	−14±4
	mdx	−15±4	−26±5	−25±4
End systolic volume	con	−36±8	−50±6	−38±6
	mdx	−36±7	−55±6	−48±9
Stroke volume	con	3±7	−1±2	0±4
	mdx	1±5	−7±5	−12±4
Ejection fraction	con	10±1	16±3	11±2
	mdx	10±1	16±1	12±4
Peak filling rate	con	−21±7	−40±4	−40±4
	mdx	−10±5	−23±5	−53±5
Peak ejection rate	con	−10±2	−6±4	−5±4
	mdx	−15±3	−10±5	−19±5
**Right ventricle**				
End diastolic volume	con	12±10	20±23	14±5
	mdx	30±12	52±25*	23±5
End systolic volume	con	5±15	60±16	39±15
	mdx	49±23*	92±14*	40±15
Stroke volume	con	16±10	−1±7	1±7
	mdx	23±8	27±10*	5±12
Ejection fraction	con	3±1	−10±3	−7±3
	mdx	−2±2	−10±2	−8±4
**Heart rate (%)**	con	19±5	9±3	5±2
	mdx	23±5	7±2	4±2

Results are presented as means ± SEM.

*(note: dobutamine stress increased mortality in older mdx mice)*.

An opposite response to dobutamine was observed in the right ventricle, with EDV and ESV increased and EF reduced (Table 2). As early as 1 month of age there was a greater increase in RV ESV in mdx mice after dobutamine compared with WT mice. By 3 months, both ESV and EDV were increased under dobutamine stress relative to WT.

### In vivo identification of myocardial fibrosis in mdx mouse hearts using late gadolinium-enhanced MRI

**Figure 4:**
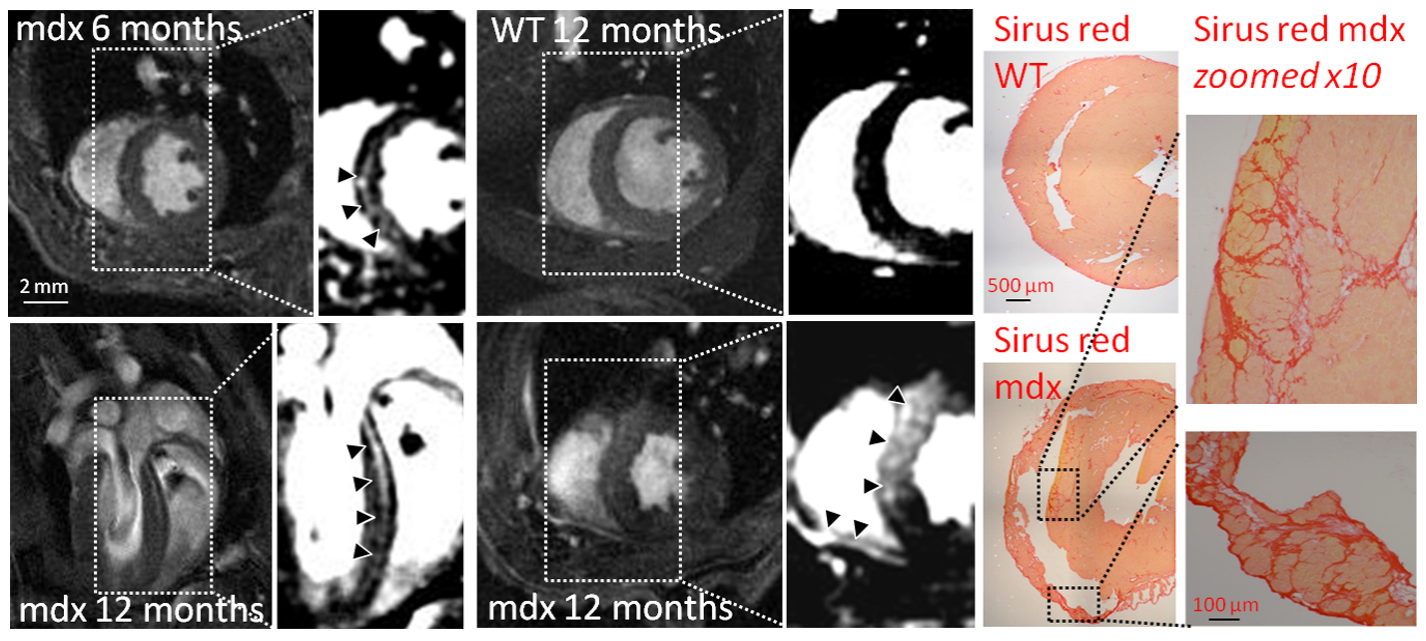
Late gadolinium enhancement in 6 and 12 month mdx mice. *Upper left;* late gadolinium enhancement in 3 of 9 mdx mice at 6 months (arrows). *Lower panel;* late gadolinium enhancement was present in the septum of all mdx mice imaged at 12 months in both long axis *(left)* and short axis *(right)* views as well as RV wall (arrows). *Upper right;* no late gadolinium enhancement was present in wild type mice (WT) at any time point. *Far right;* Sirius red staining of cryosections confirmed presence of fibrosis in the septum and RV wall of mdx mouse hearts.

**Figure 5:**
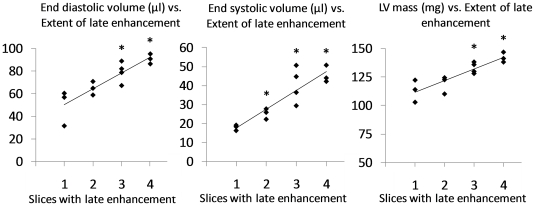
Greater cardiac remodeling in hearts with more late gadolinium enhancement. End diastolic volumes *(left)* and end systolic volumes *(middle)* and left ventricular mass *(right)* were increased in hearts with more segments showing late gadolinium enhancement.

Small regions of mid-myocardial late gadolinium enhancement were identified in the septum of four of nine mdx mice imaged at 6 months (Figure 4). By 9 months, four mice showed septal late gadolinium enhancement (not shown). At 12 months all six mdx mice displayed varying degrees of late gadolinium enhancement in the septum or RV insertion points (Figure 4). Late gadolinium enhancement was not identified in wild type control mice at any time point. The diffuse nature of the fibrosis made it difficult to accurately segment the volume of late gadolinium enhancement, but the number of imaging slices that showed enhancement could be calculated and indicated that greater cardiac remodeling and hypertrophy was present in hearts that displayed more late gadolinium enhancement (Figure 5). There also appeared to be late gadolinium enhancement in the RV wall of 12-month-old mdx mice (Figure 4). However, the spatial resolution of the imaging technique used had limited ability to visualize the thin RV wall, making the true location of the enhancement uncertain.

Tissue sections stained for sirius red confirmed the presence of fibrotic tissue within the intra-ventricular septum as well as extensive fibrosis in the right ventricular wall (Figure 4). Only small, non localized areas of fibrosis were identified in a few of the sections from the LV free wall. No fibrosis was identified in sections taken from 1-month-old mdx mice (Figure S1).

## Discussion

*In vivo* MRI of mouse models of human cardiac disease can give valuable insights into pathology and therapy. Although the mdx mouse model of muscular dystrophy has been studied using ultrasound and haemodynamic measurements [5], [8], [11], [12], [18], [19], there are limited data from MRI studies [6], [7], [13] and no reports of serial MR imaging of disease progression in the same mdx mice over time. Clinically and experimentally, cardiac MRI is more sensitive to small alterations in cardiac function than other imaging modalities, including ultrasound [22], [34], [35]. Hence, we anticipated that the application of MRI to the mdx mouse, known to have only mild cardiomyopathy when young [5], [8], [11], [12], [18], [19], may identify subtle changes previously missed by other techniques.

In agreement with previous reports [5], [6], [7], [8], [9], [10], [11], [12], standard cine-MRI did not show altered resting LV function in mdx mice until 9 months of age. However, high temporal resolution cine-MRI indicated that peak LV filling and ejection rates were lower at 3 and 6 months of age, suggesting impaired systolic and diastolic function even in young animals, as has been observed *ex vivo* in isolated, working 3-month-old mdx mouse hearts [36] and *in vivo* in 8-month-old mice [13]. Interestingly, differences in LV filling and ejection rates were not detected at 12 months, suggesting that progressive remodeling and hypertrophy may compensate for dysfunction and mask the original abnormalities at later time points. This is supported by the study of Li *et al* [7], which reported increased regional strain and torsion in the 2-month-old mdx mouse heart and reduced torsion by 10 months. All mice used in this study were male; female mice may have given different results [15].

We have reported RV dysfunction in mdx mice at 8 months that precedes LV dysfunction [13], probably owing to pulmonary hypertension and dysfunction of the diaphragm muscle [10]. Here, by performing serial imaging, we were able to determine that resting RV function was unaltered at 1 month, but abnormal as early as three months, with RV-ESV increased and RV-EF reduced. Right ventricular dilatation occurs in DMD patients at between 16 and 23 years of age [2], [26] secondary to pulmonary hypertension resulting from hypoxia-mediated constriction of pulmonary arterioles [6]. The size and complex geometry of the RV makes accurate and reproducible measurement of volume difficult when using two dimensional ultrasound or low resolution nuclear imaging methods. High resolution MRI provides the best method for *in vivo* assessment of RV function in mice [33] as it can acquire volumetric images across the entire RV, avoiding geometric calculation of volume. The ability of MRI to identify early alterations to RV function in the mdx mouse may offer a useful marker for assessing disease development and response to therapy.

Cardiac stress testing has been widely used to identify cardiac abnormalities that were undetectable at rest [19], [37], [38], [39], and can highlight ischemia-induced wall motion abnormalities, hibernating myocardium and contractile reserve [37], [38], [39]. There are several methods for performing stress testing in small animals, including intravenous infusion of increasing doses of drug until pump failure [19] and a bolus, inter-peritoneal injection of a lower drug dose that is still capable of increasing cardiac work [38], [39]. As we have shown that low dose dobutamine MRI is able to identify abnormalities in a mouse model of dilated cardiomyopathy [38], the latter method was employed here to avoid mortality that would (necessarily) compromise a serial imaging study.

Dobutamine stress testing of 1, 3 and 6 month mice reduced left ventricular EDV and ESV and increased EF and septal and free wall thickness and thickening to a similar degree in WT and mdx mice. This is the first report of stress testing in 1-month-old mdx mice, but previous studies conducted on older mice have reported reduced contractile reserve, measured using hemodynamics [8], [18], [19]. This difference may be a reflection of the high and often lethal levels of beta-adrenergic stimulation used by others. In both mdx and WT mice, peak ejection rates increased under dobutamine stress, whilst peak filling rates were reduced. It is unclear why peak filling rate would decrease under stress, but it may reflect the lower EDV of dobutamine-stimulated hearts. However, SV was not altered and the shape of the dV/dt curves showed a widening of the high velocity filling phases of the cardiac cycle with partial fusion of the early and atrial components in some mice (Figure 3).

Dobutamine stress revealed right ventricular dysfunction. To our knowledge, there are no reports of stress testing of right ventricular function in mice. We found that EDV and ESV increased and EF reduced with stress. This suggests that the LV response to dobutamine increased RV return and elevated RV pressure. We frequently observed septal bowing during peak dobutamine stress, indicative of increased RV pressures. As contractility of the RV was not elevated to the same level as the LV, the RV dilated and consequentially EF was reduced, in agreement with clinical reports [40]. As early as 1 month of age there was a greater increase in RV-ESV in mdx mice after dobutamine compared with WT. By 3 months, both ESV and EDV were increased under dobutamine stress relative to WT. However, these differences did not persist at 9 months, probably owing to the reduced response to dobutamine in 9-month-old mice. It is unclear whether this stress-induced abnormality is directly due to the effect of dobutamine on the heart (possible through stress-induced ischemic dysfunction [41]), or indirectly caused by an altered response of the diaphragm, which shows increased contractility with dobutamine [42] and could influence RV function. This stress-mediated elevation in RV-ESV is the earliest reported *in vivo* measurement of abnormal cardiac function in the mdx mouse and may indicate that, even at a very early age, pulmonary and diaphragm dysfunction can induce RV dysfunction.

Over the past decade late gadolinium-enhanced MRI has become established as one of the most useful techniques for non-invasive measurement of myocardial viability [43], [44], [45], [46], [47], [48]. In recent years late gadolinium-enhanced MRI has been able to detect fibrosis in patients with hypertrophic [49] and dilated [50] cardiomyopathy, systemic vasculitus [43], arrhythmogenic right ventricular disease [45] and DMD [25], [26], [27] and Becker muscular dystrophy [22], [27].

Late gadolinium-enhanced MRI methods have been developed for imaging mice post myocardial infarction [31], [51], but to date no studies of congenital cardiomyopathies have been reported. We detected late gadolinium enhancement in 3 out of 9 mdx mice of 6 months of age. By 12 months of age, all mdx mice displayed some late gadolinium enhancement. No late gadolinium enhancement was identified in control mice. Similar to clinical reports [24], [28], the extent of late gadolinium enhancement correlated with the degree of cardiac impairment and remodeling. Histology has shown that fibrosis develops in the hearts of mdx mice from 6 months [12], the extent related to impairment of cardiac function [5]. The *in vivo* methods presented here will be useful in assessing the efficacy of gene therapy studies in the mdx mouse [4], [6], [11], [16], [19].

This study highlights the power of comprehensive and serial non-invasive imaging for assessment of cardiac function in experimental models of human disease. The detection of abnormalities in young mice gives important information regarding the speed of disease progression and response to therapy. To our knowledge, there have been no reports of cardiac MR imaging in mice as young as one month. Many transgenic animals develop cardiac abnormalities at an early age and may not survive adolescence. The results presented here demonstrate that it is feasible to image one-month-old mice and detect early alterations in function that could be valuable in characterization of animal models of human cardiac disease. Further, we show that small animal MRI measurements are directly comparable to those made in patients, increasing the relevance of pre-clinical data and speeding translation of novel therapies.

## Supporting Information

Figure S1 **Sirius red staining of cryosections from 1-month-old mdx mice (n = 3) did not detect any fibrosis in the LV or RV walls.**
(TIF)
