# Supplementary figures and images for: In vivo MRI Characterization of Progressive Cardiac Dysfunction in the mdx Mouse Model of Muscular Dystrophy
Source: PLoS One. 2012 Jan 3;7(1):e28569. doi: 10.1371/journal.pone.0028569 (PMC3250389; doi:10.1371/journal.pone.0028569)

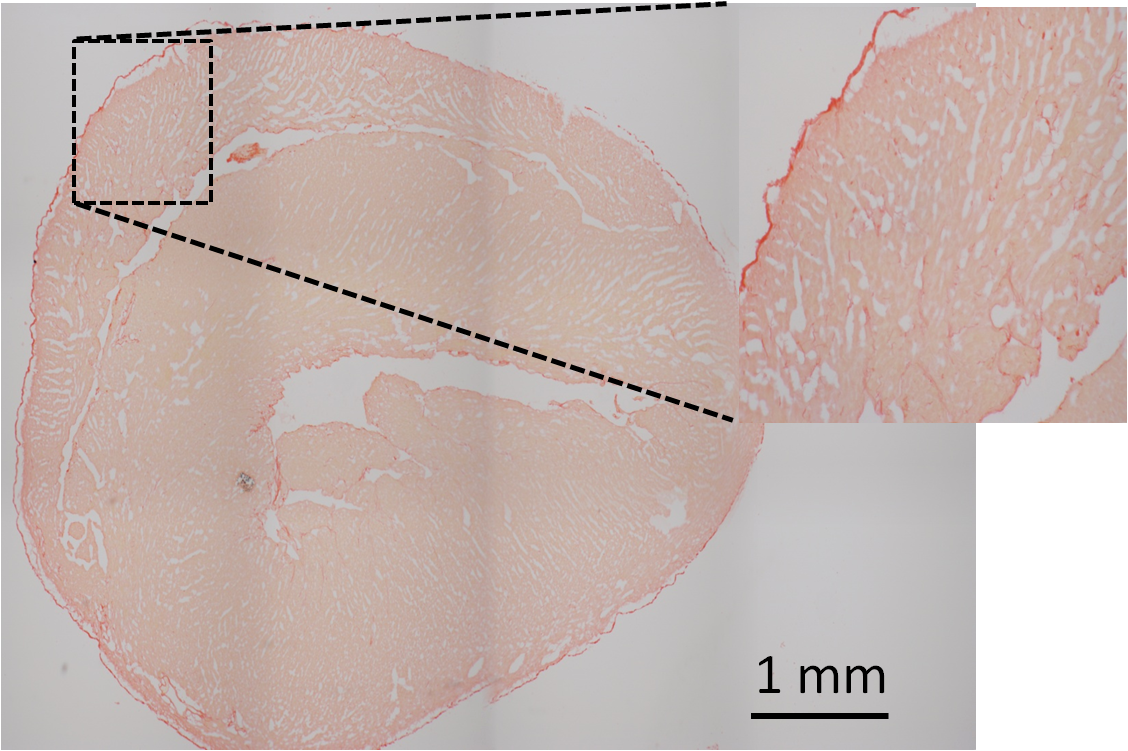

Supplement: Figure S1 — Sirius red staining of cryosections from 1-month-old mdx mice (n = 3) did not detect any fibrosis in the LV or RV walls. (TIF) [file pone.0028569.s001.tif]
